# Genomic and Physiological Characterization of Bacilli Isolated From Salt-Pans With Plant Growth Promoting Features

**DOI:** 10.3389/fmicb.2021.715678

**Published:** 2021-09-13

**Authors:** Claudia Petrillo, Stefany Castaldi, Mariamichela Lanzilli, Matteo Selci, Angelina Cordone, Donato Giovannelli, Rachele Isticato

**Affiliations:** ^1^Department of Biology, University of Naples Federico II, Complesso Universitario Monte S. Angelo, Naples, Italy; ^2^Institute of Biomolecular Chemistry (ICB), CNR, Pozzuoli, Italy; ^3^National Research Council – Institute of Marine Biological Resources and Biotechnologies (CNR-IRBIM), Ancona, Italy; ^4^Department of Marine and Coastal Science, Rutgers University, New Brunswick, NJ, United States; ^5^Department of Marine Chemistry & Geochemistry, Woods Hole Oceanographic Institution, Woods Hole, MA, United States; ^6^Earth-Life Science Institute, Tokyo Institute of Technology, Tokyo, Japan; ^7^Interuniversity Center for Studies on Bioinspired Agro-Environmental Technology (BAT Center), Portici, Italy

**Keywords:** spore-forming bacteria, biocontrol agents, halophiles, plant-growth-promoting bacteria, genome mining, Bacilli

## Abstract

Massive application of chemical fertilizers and pesticides has been the main strategy used to cope with the rising crop demands in the last decades. The indiscriminate use of chemicals while providing a temporary solution to food demand has led to a decrease in crop productivity and an increase in the environmental impact of modern agriculture. A sustainable alternative to the use of agrochemicals is the use of microorganisms naturally capable of enhancing plant growth and protecting crops from pests known as Plant-Growth-Promoting Bacteria (PGPB). Aim of the present study was to isolate and characterize PGPB from salt-pans sand samples with activities associated to plant fitness increase. To survive high salinity, salt-tolerant microbes produce a broad range of compounds with heterogeneous biological activities that are potentially beneficial for plant growth. A total of 20 halophilic spore-forming bacteria have been screened *in vitro* for phyto-beneficial traits and compared with other two members of *Bacillus* genus recently isolated from the rhizosphere of the same collection site and characterized as potential biocontrol agents. Whole-genome analysis on seven selected strains confirmed the presence of numerous gene clusters with PGP and biocontrol functions and of novel secondary-metabolite biosynthetic genes, which could exert beneficial impacts on plant growth and protection. The predicted biocontrol potential was confirmed in dual culture assays against several phytopathogenic fungi and bacteria. Interestingly, the presence of predicted gene clusters with known biocontrol functions in some of the isolates was not predictive of the *in vitro* results, supporting the need of combining laboratory assays and genome mining in PGPB identification for future applications.

## Introduction

In the past decades, social concern about the environmental effects of the uncontrolled use of chemical pesticides, fertilizers, and herbicides in the agricultural field has risen considerably. The use of chemicals for the protection and enhancement of crops has led to several negative consequences: the formation of stable phytopathogenic variants, the reduction in the number of beneficial microorganisms, and the accumulation of toxic substances in soils and aquatic ecosystems (Reddy et al., 2009; Pertot et al., 2017). Given the increased global demand for crop production, researchers and industries are seeking new, more sustainable and greener approaches to pesticides and fertilizers (Glick et al., 2007). In this framework, the use of microorganisms known as Plant-Growth-Promoting Bacteria (PGPB) for crop production appears to be a promising alternative. PGPB improve crop fitness and yields both, through direct and indirect mechanisms. Direct mechanisms include the promotion of alternative nutrient uptake pathways, through the solubilization of phosphorus, the fixation of atmospheric nitrogen, the acquisition of iron by siderophores, and the production of growth hormones and molecules like vitamins, amino acids, and volatile compounds (Babalola, 2010). Indirect mechanisms instead, include the prevention or reduction of the damage induced by phytopathogens through the production of different classes of antimicrobial compounds such as hydrolytic enzymes that can lyse a portion of the cell wall of many pathogenic fungi (Jadhav et al., 2017).

The work presented here is part of a wide study aimed at identifying and selecting halophilic *Bacilli* with potential applications as biofertilizers or biocontrol agents. For this purpose, samples from the rhizosphere of the nurse plants *Juniperus sabina* and nearby soils were collected from salt-pans (Castaldi et al., 2021). Nurse plants, such as *J. sabina*, exert beneficial effects on their surrounding ecosystem, facilitating the growth and development of other plant species. This positive effect is in part due to the plant influence on the composition of soil microbial communities, generally selecting for microorganisms capable of mineralizing nutrients, enhancing soil fertility, and thus promoting plant growth and health (Hortal et al., 2013; Goberna et al., 2014; Rodríguez-Echeverría et al., 2016). For this reason, the nurse-plants rhizosphere and relative surrounding soil are a useful source of PGPB. In addition, bacteria growing in extreme environments, like salt-pans, have developed complex strategies to survive harsh conditions, which include the production of an array of diverse compounds, such as antioxidant pigments, lytic enzymes, and antimicrobial compounds, making them interesting biotechnological targets (Anwar et al., 2020). Among the PGPB, bacteria belonging to the *Bacillus* genus are of particular interest given their resistance to stressful environments and conditions due to their capacity of producing spores (Pesce et al., 2014), together with the ability to release a broad spectrum of secondary metabolites, the easy genetic manipulation, and the great ability to colonize plant surfaces (Kumar et al., 2011). In addition, the effectiveness of halo-tolerant *Bacillus* spp. to increase the growth of various crops under salt stress conditions has been widely reported (Shultana et al., 2020). Recently, we have identified and characterized PGPB *Bacillus* strains isolated from the rhizosphere of *J. sabina* (Castaldi et al., 2021). The two strains, named as *Bacillus* sp. RHFS10 and *Bacillus* sp. RHFS18, emerged for their promising PGP traits. These strains produce siderophores and solubilize phosphorus, enhancing plant nutrients uptake, and secrete indoleacetic acid (IAA), a phytohormone playing a key role in both root and shoot development. Additionally, both isolates showed a strong biocontrol activity, inhibiting the fungal phytopathogen *Macrophomina phaseolina* growth (Castaldi et al., 2021).

Here, we present the results of the screening of 20 halophilic *Bacilli* isolated from salt-pan sand samples. All the strains were characterized for PGP traits and five strains emerged for their high potentiality as biofertilizers and biocontrol agents. Comparative genomic analysis of the five sand strains and the previously characterized rhizospheric strains RHFS10 and RHFS18 revealed the presence of known genes involved in plant growth promotion and protection, sustaining, in part, the activities observed *in vitro*. Overall, this work suggests a strategy for the selection of potential PGP candidates belonging to *Bacillus* genus using combined *in silico* and *in vitro* approaches.

## Materials and Methods

### Isolation of Bacteria

*Bacillus* strains used in this study were isolated from sand samples collected in the proximity of *J. sabina* plants growing in the salt-pans of Formentera (Spain). Sand samples were heat-treated at 80°C, for 15min to kill vegetative cells and select for spore-forming bacteria, and 1g of sample was suspended in 9ml of TY broth (10g/L tryptone, 5g/L yeast extract, and 8g/L NaCl) and 10-fold serial dilutions placed on TY plates (Cangiano et al., 2010). After 4–5days of incubation at 30±1°C, colonies were recovered and streaked on fresh TY plates, and pure cultures stored at −80°C into glycerol stocks (Giglio et al., 2011).

### Phenotypic Characterization and Growth Conditions

The phenotypic variants of isolated strains were determined by visual inspection. The facultative anaerobic growth was determined using the AnaeroGen sachets (Unipath Inc., Nepean, Ontario, Canada) placed in a sealed jar with bacteria streaked on TY agar plates and incubated at 37°C for 3days. To confirm the sporulation ability, the bacteria were grown in Difco sporulation medium (8g/L Nutrient broth No. 4, 1g/L KCl, 1mM MgSO_4_, 1mM Ca(NO_3_)_2_, 10μM MnCl_2_, and 1μM FeSO_4_, Sigma-Aldrich, Germany) at 37°C for 30–48h, and the presence of spores was checked by light microscopy. Salt, pH, and temperature tolerance were determined as follows: about 50μl of culture of each isolate grown in TY broth for 6h at 37°C (10^7^ cells/ml) were transferred to individual tubes containing 5ml of TY broth with different pH (2.0, 4.0, 6.0, 7.0, 8.0, 10.0, and 12.0) or NaCl concentration (0, 5, 10, 13, 15, and 18%) and left to grow at 37°C with agitation (Cangiano et al., 2014) . The temperature tolerance of isolates was tested incubating the cultures at 37 (control), 4, 15, 25, 50, and 60°C. The growth (+) or no growth (−) in comparison with the controls after 24–48h was recorded.

### Plant Growth-Promoting Traits

#### Phosphate Solubilization

The phosphate solubilization activity was evaluated by spot inoculation of 3μl of the freshly grown bacterial culture (10^7^ cells/ml) onto Pikovaskya’s agar medium (Pikovskaya, 1948). The plates were incubated at 28°C for 10days. The formation of transparent zones around the bacterial colonies indicates a positive result (Schoebitz et al., 2013).

#### Siderophores Production

The siderophores production was determined by the Chrome Azurol S (CAS) assay as described by Pérez-Miranda et al. (2007). Three milliliter of freshly-grown bacterial cultures was spot-inoculated on CAS agar plates and incubated at 28°C. The formation of a yellow-orange halo zone around the bacterial colony was a positive indicator of siderophore production and the halo zone diameters were measured after 4days of incubation.

#### Indoleacetic Acid Detection

The indoleacetic acid production was measured as described by Etesami et al. (2014), with some modifications. Briefly, each strain was cultured in 10ml of TY broth at 37°C for 4days with shaking at 150rpm. Following growth, 1ml of bacteria supernatant was mixed with 2ml of Salkowski reagent (0.5M FeCl_3_ in 35% HClO_4_ solution), and the solution was vortexed and incubated at room temperature for 30min. The formation of pink color was considered a positive reaction (Damodaran et al., 2013). Quantitative estimation of IAA (μg/ml) was obtained by recording spectroscopic absorbance at 535nm using a standard curve prepared separately with pure IAA (Sigma) in the range 0–100μg/ml (Gordon and Weber, 1951). Sterile TY medium was used as control.

#### Biofilm Production and Swarming Motility

To detect the ability to produce biofilm, bacterial isolates were grown in 24-well culture plates in TY broth for 48h without agitation at 37°C in according to O’Toole (2011). Then, the supernatant was discarded, adhered cells were rinsed three times with distilled water and 1ml of a 0.1% crystal violet (CV) solution was added to stain the adhered biomass. Plates were incubated for 30min at room temperature, washed carefully three times with distillated water and patted dry. Dye attached to the wells was extracted with 1ml of 70% ethanol and quantified at an absorbance of 570nm. Data were normalized by total growth estimated by OD600 nm, and the experiment was performed in triplicate.

Swarming motility was tested according to the method adopted by Adler (1966). TY agar 0.7% plates were spot inoculated with 3μl of the freshly grown bacterial culture (10^7^ cells/ml). After an overnight incubation at 37°C, the swarm diameters were measured.

### Whole-Genome Sequencing of the Selected PGPB

DNA extraction was performed using the DNeasy PowerSoil kit (Qiagen, Hilden, Germany) according to the manufacturer’s instructions. Genome sequencing was performed by MicrobesNG (Birmingham, United Kingdom) with the genomic DNA library prepared using the Nextera XT library prep kit (Illumina) following the manufacturer’s protocol. Libraries were sequenced on the Illumina HiSeq using a 250bp paired-end protocol. Reads were adapted and trimmed using Trimmomatic 0.30 with a sliding window quality cutoff of Q15 (Bolger et al., 2014) and the *de novo* genome assembly was carried out with SPAdes (version 3.7) *via* MicrobesNG. Genomes were annotated using Prokka (Seemann, 2014). Biosamples accession numbers for strains RHFB, RHF2, RHF6, RHF12, RHF15, RHS10, and RHFS18 are, respectively: SAMN17389615, SAMN17389609, SAMN17389610, SAMN17389612, SAMN17389613, SAMN17389611, and SAMN17389614. MIGS compliant details regarding each genome are available in the Supplementary Table S1.

Average Nucleotide Identity (ANI) values between the sequenced genomes and the closest bacterial species identified from the 16S rRNA phylogenetic analysis (see below) were obtained using the OrthoANI algorithm of EZBioCloud (Yoon et al., 2017). An ANI similarity of 95% was considered as a cut-off for species delineation.

### Phylogenetic Analysis

The 16S rRNA genes were extracted from the sequenced genomes using Anvi’o v2.3.3 (Eren et al., 2021). and compared to 76 reference 16S rRNA genes from closely related strains identified using the Genome Taxonomy Database (GTDB)^1^ taxonomy and retrieved from the NCBI database. All sequences were aligned using Seaview 4.4.0 software (Corrado et al., 2021), and the phylogenetic tree was constructed using the Maximum-likelihood algorithm with model GTR+I+G4. Statistical support was evaluated by the approximate likelihood-ratio test (aLRT) and is shown at the corresponding nodes of the tree. *Clostridium difficile* is used as an outgroup to root the tree.

### Evaluation of Potential Biocontrol Activity

Isolated bacterial strains were tested *in vitro* for growth inhibitory activity against phytopathogenic fungi and bacteria are listed in Table 1. The phytopathogenic fungi are deposited in the fungal culture collection of the Plant Pathology Department of the University of Buenos Aires (FAUBA, Argentina) and were kindly supplied by Marcelo Anibal Carmona (Facultad de Agronomía, Cátedra de Fitopatología, Universidad de Buenos Aires, Buenos Aires, Argentina), except for *Stemphylium vesicarium*. All the fungi were stored on Potato Dextrose Agar (PDA) in Petri dishes. Dual-culture plate method was carried out to detect the antifungal activity in accordance with Xu and Kim (2014). Briefly, fungal plugs of 6mm×6mm diameter were placed at the center of PDA plates and 5μl of bacterial strains grown overnight in TY broth were placed on the opposite four sides of the plates 1.5cm away from the fungal disc. This method was repeated for each fungus. Controls consisted of plates containing the fungal plugs alone. All plates were incubated at 28°C for 5–7days. The antagonism activity against bacterial phytopathogens was performed as described in Li et al. (2020) with some modifications. Bacterial pathogens were streaked on TY plates and incubated at 25°C overnight. Single colonies were suspended in TY broth and incubated at 25°C. Approximately 1×10^−6^CFU/ml were mixed with melted 0.8% TY agar medium before pouring plates. After solidification, 5μl of bacterial isolates solution (OD_600_=1.0) was spot inoculated onto the plates and incubated at 28°C for 48h, before measuring the diameters of inhibition halos. All experiments were performed in triplicate.

**Table 1:** List of the phytopathogenic fungi and bacteria used in this study.

Pathogen type	Species	Strain	Provenience	Host plant
Fungi	*Macrophomina phaseolina*	2,012,013-1	Argentine	Soy
	*Colletotrichum truncatum*	17-5-5	Argentine	Soy
	*Drechslera teres*	FT	Argentine	Barley
	*Cercospora nicotianae*	Ck_2017_B35	Bolivia	Soy
	*Stemphylium vesicarium*		Italy	Pear
Bacteria	*Pseudomonas tolaasii*	2,192	-	Mushroom
	*Pseudomonas syringae pv tabaci*	ICMP 2706	-	Tobacco
	*Pseudomonas syringae pv panici*	ICMP 3955	-	Rice
	*Pseudomonas caryophylli*	NCPPB349	Italy	Carnations
	*Pseudomonas syringae pv syringae*	B475	-	Mango
	*Pseudomonas syringae pv japonica*	ICMP 6305	-	Wheat
	*Pseudomonas syringae pv papulans*	Psp26	-	Apple

### Identification of Biosynthetic Gene Clusters

Obtained genomes were analyzed by antiSMASH 5.0 (Blin et al., 2019) and BAGEL 4 (van Heel et al., 2018) to identify biosynthetic gene clusters (BCGs) of potential antimicrobial compounds such as non-ribosomal peptide synthetases (NRPSs), polyketide synthases (PKSs), post-translationally modified peptides (RiPPs), hybrid lipopeptides (NRPS-PKS) and bacteriocins. Biosynthetic Gene Clusters that shared less than 70% amino acid identity against known clusters were regarded as novel.

## Results and Discussion

### Isolation and Characterization of Spore-Forming Plant-Growth-Promoting Bacteria

Spore-forming bacteria were specifically isolated from sand samples collected from gaps among nurse plants, belonging to the genus *J. sabina*, in salt-pans as described in the Materials and Methods section. Based on morphological characteristics, a total of 20 isolates were selected and preliminarily characterized for growth properties (Supplementary Table S2). All the strains can be classified as facultative anaerobic, mesophiles and moderate halophiles, excluding RHF5 strain, which survives up to 60°C and strain RHFB unable to grow at temperature and salt concentration higher than 37°C and 5% NaCl, respectively (Ventosa et al., 1998; Schiraldi and De Rosa, 2016).

To identify potential PGPB, the 20 strains were evaluated *in vitro* for physiological traits associated with plant growth enhancement and biocontrol ability (Table 2). Strain performance was compared with those of two promising PGPB, RHFS10, and RHFS18 strains, belonging to the *Bacillus* genus and isolated from *J. sabina* rhizosphere of the same collection site (Castaldi et al., 2021) and proposed as biocontrol agents for their antagonistic activity against the phytopathogen *M. phaseolina*. Most of the new strains displayed root-colonization phenotypes since able to surface spread by swarming and to form biofilms (Amaya-Gómez et al., 2020), while only five were found either positive to both solubilization of phosphate, indoleacetic acid (IAA), and siderophore production. Strains RHF6, RHF15, and RHFB showed a better performance than when compared against the already characterized rhizobacteria strains RHFS10 and RHFS18, confirming that the microenvironments created under or nearby nurse shrubs are a promising source of PGPB (Rodríguez-Echeverría et al., 2016). All bacterial isolates were tested for *in vitro* activities of their extracellular hydrolytic enzymes (lipase, protease, amylase, xylanase, and cellulase) usually associated with biocontrol activity (Pal and McSpadden Gardener, 2006). As reported in Table 2, the highest hydrolytic activity was observed for RHF12, RHF15, and RHFB strains, comparable with that exerted by rhizosphere strains RHFS10 and RHFS18.

**Table 2:** Summary of plant growth-promoting and biocontrol traits exhibited by 20 spore-forming bacteria isolates.

Strain	PGPB activities					Hydrolytic activities				
	Biofilm (OD_570_)	Swarming	PVK	IAA (μg/ml)	Siderophores	Lipase	Protease	Amylase	Xylanase	CMC
RHF1	−	++	++	−	+	−	++	++	+	++
**RHF2**	0.2	+	+	18	+	−	+	+	+	+
RHF3	−	−	−	−	−	+	++	++	+	−
RHF4	−	+	−	−	+	+	++	++	−	+
RHF5	0.2	−	−	2	−	−	+	++	−	−
**RHF6**	0.3	+	++	31	++	−	+	+	+	++
RHF7	0.4	−	−	−	−	−	+	+	−	−
RHF8	0.6	++	−	6	−	−	++	++	++	−
RHF9	−	−	+	3.2	−	−	++	++	−	−
RHF10	−	−	−	4	−	−	++	+	+	+
RHF11	0.2	+	−	−	−	−	+	+	+	−
**RHF12**	0.7	++	+	25	++	−	++	++	++	++
RHF13	−	++	++	3	+	+	−	++	+	++
RHF14	−	−	−	−	−	+	+	+	+	−
**RHF15**	0.6	++	++	23	++	+	++	++	++	++
RHF16	−	−	−	−	−	+	+	+	−	−
RHF17	0.5	++	+	−	+	+	+	+	++	+
**RHFB**	0.3	+	++	32	++	++	++	++	++	+
RHFE	−	−	−	−	−	+	+	+	−	−
RHFL	0.3	−	−	−	−	−	+	+	−	−
**RHFS10** ^1^	0.3	++	+	12	++	++	++	++	++	++
**RHFS18** ^1^	0.5	+	+	12	++	+	++	++	++	++

No activity (−), halo or colony diameter <5mm (+), halo or colony diameter ≥5mm (++), halo or colony diameter 10mm (+++). Data are represented by means of at least three replicates±SE at *p*≤0.05 using LDS. The strains selected for further studies are indicated in bold. PVK, Pikovskaya; IAA, indoleacetic acid; and CMC, carboxymethylcellulose.

1 Available from Castaldi et al. (2021).

Based on these results reported in Table 2, seven strains were selected for whole-genome sequencing analysis. All selected strains were able to solubilize phosphate with efficiency higher than the other ones and to produce Biofilm, IAA, and siderophores. Further, strains RHF12, RHF15, RHFB, and RHFS18 emerged for their strong hydrolytic potential, often associated to biocontrol activity (Castaldi et al., 2021), while strain RHF6 showed the ability to growth up to 13% NaCl, showing the best salt tolerance (Supplementary Table S2).

### Genome Sequencing and Phylogenetic Analysis

**Figure 1:**
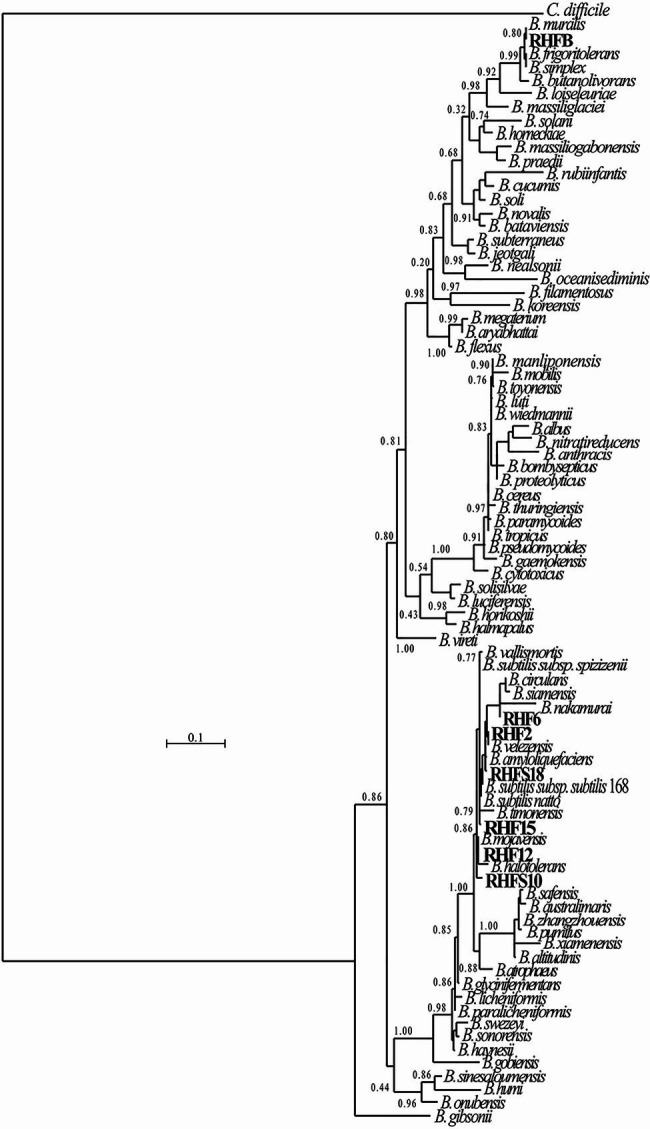
Phylogenetic tree of the spore-forming bacteria isolated from salt-pans. The phylogenetic tree was constructed using the Maximum-likelihood algorithm with model GTR+I+G4, based on 16S rRNA gene sequences. The gene sequences of the isolated bacteria were aligned to reference bacteria belonging to the *Bacillaceae* family according to Genome Taxonomy Database (GTDB). Node support represents the approximate likelihood-ratio test (aLRT) and is shown at the corresponding node of the tree. *Clostridium difficile* is used as an outgroup.

The obtained genomes had coverage of ~30×, with a variable number of contigs between 40 and 1,105 for RHF15 and RHFS18, respectively (Table 3). The genome of strain RHFS18 was particularly fragmented, and repeated sequencing of the same strain did not yield improved assembly suggesting that the results are not dependent on a low-quality sequencing library. The obtained genomes are approximately 4.0Mbp long except for RHFB’s genome, being the longest (5.6Mbp) and the one with the highest number of predicted protein coding sequences compared to the others. Taxonomic identification of the strains was based on the phylogenetic analysis of the 16S rRNA sequence as well as the whole genome Average Nucleotide Identity. All the isolates were identified as members of the genus *Bacillus* (Figure 1) with six strains out of seven clustering into the same clade, and only strain RHFB falling in a different clade. The phylogenetic divergence observed for RHFB from the other strains agrees with the observed differences in physiological traits for this strain (Supplementary Table S3). Since most *Bacillus* species are phylogenetically close, 16S rRNA analysis is not always exhaustive to obtain an unambiguous assignment (Rooney et al., 2009). To overcome this issue and classify the strains at the species level, whole genome ANI was used (Table 4). Strain RHFB exhibited 96.95% ANI against the genome of the closest relative *Brevibacterium frigoritolerans* and was therefore identified as a *B. frigoritolerans* species. Strain RHF2 was identified as *Bacillus subtilis*, based on 99.96% ANI score. Strains RHF6 and RHFS18 were classified as members of the *Bacillus amyloliquefaciens* species, exhibiting 99.26 and 98.36% ANI, respectively. Strain RHF12 was identified as *Bacillus halotolerans*, based on 98.04% ANI score, while RHF15 was classified as *Bacillus gibsonii*, showing 99.6% ANI score. As shown in Table 4, RHFB, RHF12, and RHFS18 strains were univocally matched with the same species, while for RHF2, RHF6, and RHF15 strains the two analyses returned different results. This mismatch between the two methods of classification is due to the poor discrimination between closely related species of the *Bacillus* genus due to their high morphological, biochemical, and genetic similarities (Celandroni et al., 2019). Since taxonomy annotations based on genetic markers, such as the 16S rRNA gene, can give variable results depending on the strain, ANI-based classification has been preferred in this study when showing ANI scores ≥95% (Jain et al., 2018). Based on this, RHF2, RHF6, and RHF15 were identified as *B. subtilis*, *B. amyloliquefaciens*, and *B. gibsonii*, respectively (Table 4). Only strain RHFS10 could not be classified at the species level due to the low ANI score (93.48%) when compared with the closest relative *Bacillus vallismortis* and it was classified as *Bacillus* sp. RHFS10 (Table 3). Further analysis will be required to fill this classification gap.

**Table 3:** General features of the assembled genomes.

Analysis statistics	Strains						
	RHFB	RHF2	RHF6	RHF12	RHF15	RHFS10	RHFS18
Size (bp)	5,648,757	4,003,762	4,066,378	4,096,200	4,232,838	4,254,653	3,936,406
Number of contigs	158	52	156	280	40	46	1,105
Mean GC content (%)	40.57	43.74	46.3	44.01	43.39	43.95	46.14
CDS	5,413	3,988	3,901	3,997	4,282	4,182	3,87
N50	187,761	413,219	584,325	60,229	2,184,724	1,139,270	6,179
N75	82,022	306,766	292,476	34,071	1,049,735	348,257	3,118
L50	11	3	2	19	1	2	176
L75	21	6	4	42	2	4	397

**Table 4:** Classification of the seven selected strains.

	16S rRNA similarity	ANI (best score)
RHFB	*B. frigoritolerans* (100%)	*B. frigoritolerans* (96.95%)
RHF2	*B. velezensis* (99.87%)	*B. subtilis* 168 (99.96%)
RHF6	*B. velezensis* (100%)	*B. amyloliquefaciens* (99.26%)
RHF12	*B. halotolerans* (98.51%)	*B. halotolerans* (98.04%)
RHF15	*B. subtilis* (100%)	*B. gibsonii* (99.6%)
RHFS10	*B. halotolerans* (97.5%)	*B. vallismortis* (93.48%)
RHFS18	*B. amyloliquefaciens* (100%)	*B. amyloliquefaciens* (98.36%)

The 16S rRNA similarity and ANI score against the closest relative identified from the phylogenetic analysis are reported for each isolate.

### Environmental Adaptation to Halophilic Conditions

**Figure 2:**
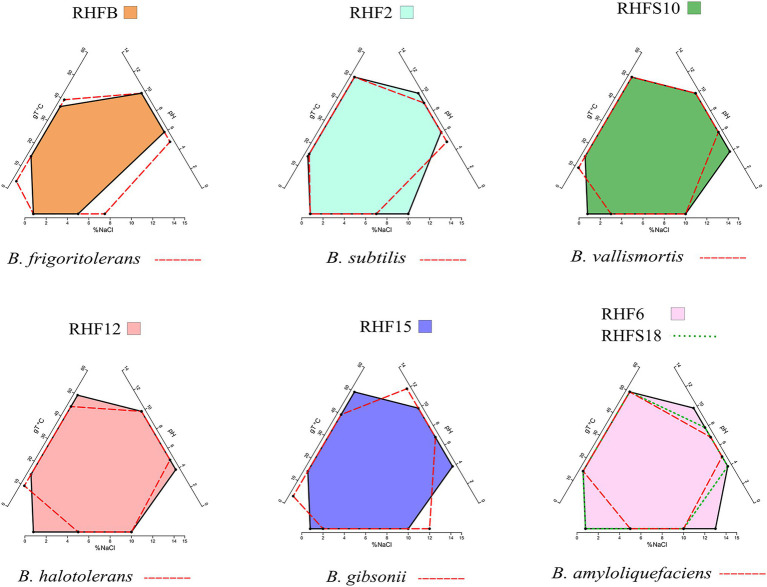
Phenotypic plasticity of the salt-pan isolates. Multivariate polygons plots (Giovannelli et al., 2021, in preparation) showing the growth temperature (gT°C), pH, and salinity (% NaCl) boundaries observed for the seven isolates (polygons) and the range for the closest relative identified by ANI (red dashed lines). Each edge represents the range for the specific variables projected onto the axis. More information about polygons plot can be found at https://giovannellilab.github.io/polygonsplot/.

The phenotypic plasticity of the salt-pans isolates was investigated by comparing their growth parameters against the closest *Bacillus* species identified by the ANI analysis (Table 4). Temperature, pH, and salinity ranges required for growth were evaluated. These parameters are useful to identify distinct phenotypic strategies used by microorganisms to better adapt to environmental conditions (Agrawal, 2001). As expected, taxonomically closer strains showed small differences when compared with each other or with their representative species (red dashed lines in Figure 2). As already highlighted by the phylogenetic analysis, *B. frigoritolerans* RHFB strain presented a diverging phenotype, especially considering the lower salt tolerance compared to the other isolates. Interestingly, some strains, like *B. halotolerans* RHF12, *B. gibsonii* RHF15, and *Bacillus* sp. RHFS10, showed identical growth properties even though belonging to three different *Bacillus* species (Figure 2), while strains of the same species, like *B. amyloliquefaciens* RHF6 and RHFS18, exhibited different adaptations to NaCl concentration and pH range. Moreover, *B. amyloliquefaciens* RHF6 like *B. subtilis* RHF2 were able to grow at higher salt concentrations than their representative species, suggesting an adaptive phenotypic variation to the high salinity condition of salt-pans.

### Analysis of Potential PGP and Biocontrol Traits

**Figure 3:**
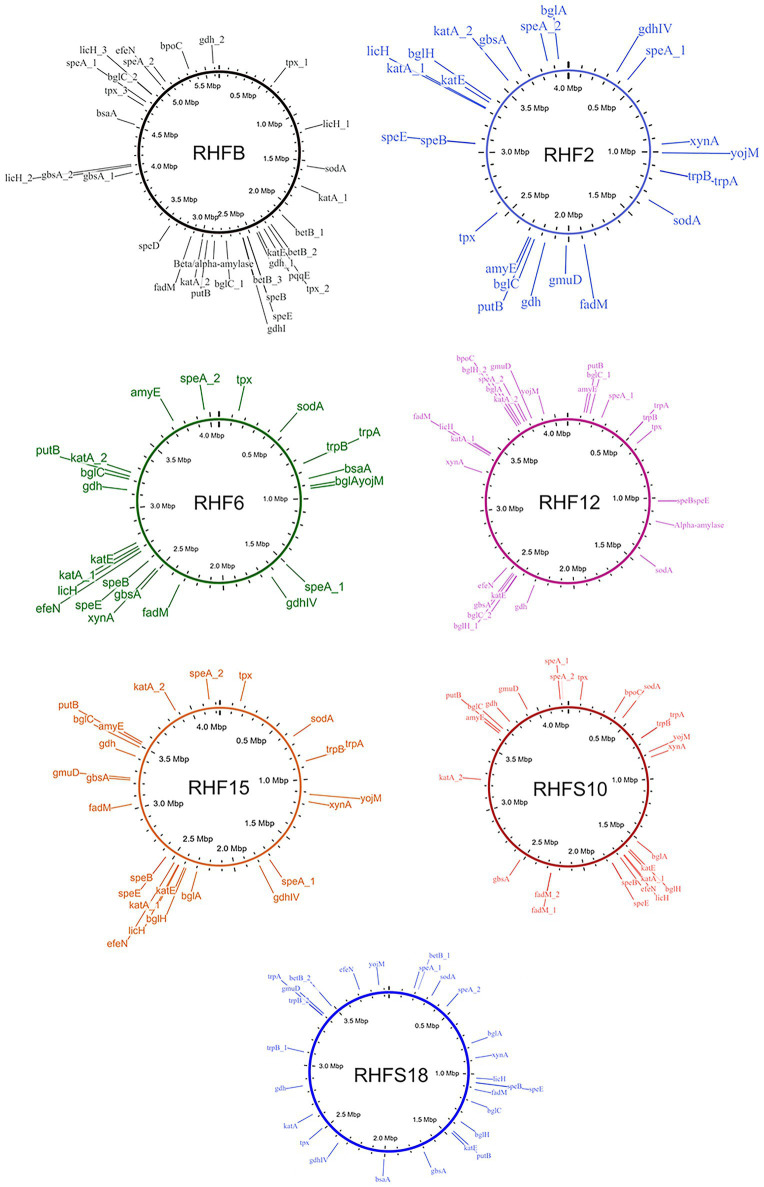
Whole genome representations of the seven isolates showing the location of the identified PGP trait genes.

To confirm the *in vitro* PGP characterization of the isolates, a prediction of the genes (Figure 3) and proteins (Table 5) involved in biocontrol activity and plant growth promotion was performed. The analyses identified genes that can be attributed to the strains ability to improve nutrient availability, suppress pathogenic fungi, and resist oxidative stress and quorum sensing in all analyzed genomes. For instance, the genome of most of the seven strains included the pyrroloquinolone quinone synthase (*pqq*) and the dependent glucose dehydrogenase (*gdh*) genes, involved in mineral phosphate solubilization as well as antifungal activities and systemic resistance induction. Interestingly, both isolates *B. amyloliquefaciens* RHF6 and RHFS18 did not carry the cofactor *pqq* gene cluster, suggesting that other mechanisms could co-exist (Table 2). IAA is one of the most common and effective plant-growth hormones. Besides plants, most rhizobacteria can produce and secrete IAA, increasing the growth and the yield of crops (Bunsangiam et al., 2019). All the strains produced Tryptophan-2-monooxygenase and Indole-3-acetamide hydrolase, able to convert Tryptophan in Indole-3-acetamide and then in IAA, respectively (Bunsangiam et al., 2019). The presence of other tryptophan synthases orthologs (subunits a and b) in all the analyzed genomes suggests alternative IAA biosynthesis pathways potentially involving different intermediates. This hypothesis is supported by the observation that *B. frigoritoleran*s RHFB, one of the best IAA producers among the isolated PGPB, possessed the indole-3-pyruvate decarboxylase, a key enzyme of another Trp-dependent pathway for IAA production (Sitbon et al, 2000).

**Table 5:** Plant-Growth-Promoting traits-associated proteins identified in the proteome of the selected strains and their abundance.

PGP Trait	Protein	RHFB	RHF2	RHF6	RHF12	RHF15	RHFS10	RHFS18
Phosphate solubilization	Pyrroloquinoline quinone	1	1	0	1	1	1	0
	Glucose 1-dehydrogenase	2	2	2	2	2	2	2
Nitrogen fixing	Nitrogenases	6	6	4	6	6	6	2
Nitric oxide synthesis	Copper-containing nitrite reductase	1	2	1	3	2	2	1
IAA biosynthesis	Indole-3-pyruvate decarboxylase	1	0	0	0	0	0	0
	Tryptophan 2-monooxygenase	4	2	1	2	2	3	2
	Tryptophan synthase (subunit a and b)	6	7	6	7	7	5	7
	Tryptophan aminotransferase	0	0	0	0	0	0	0
	Tryptophan decarboxylase	0	0	0	0	0	0	0
	Indole-3-acetamide hydrolase	0	0	0	0	0	0	0
Putrescine and Spermidine-related production	Arginine decarboxylase	3	2	2	2	2	2	2
	Agmatine ureohydrolase	1	1	1	1	1	1	2
	Ornithine decarboxylase	0	0	0	0	0	0	0
	SAM decarboxylase	1	1	1	1	1	2	1
	Spermidine synthase	1	1	1	1	1	1	1
ACC deaminase activity	ACC deaminase	2	2	3	1	2	1	3
	D-cysteine desulfhydrase	1	0	1	0	0	0	1
Antioxidant activity	Peroxidases	9	10	4	9	9	8	4
	Catalases	10	12	11	11	12	11	8
	Superoxide dismutase	7	5	5	6	5	5	5
	Glutathione peroxidase	1	1	1	1	2	1	1
	Glutathione reductase	0	0	0	0	0	0	0
	Glutathione S-transferase	2	5	2	2	5	2	3
Abiotic stress	Choline dehydrogenase	0	1	1	2	2	1	1
	Betaine-aldehyde dehydrogenase	5	2	2	2	2	2	2
	Proline dehydrogenase	2	3	2	2	2	2	2
Cell wall and degrading	β-Glucosidase	1	3	2	5	3	3	3
	α-Glucosidase	3	4	4	3	4	4	2
	Endo-1,4-β-xylanase	5	7	7	4	5	7	9
	Glucoamylase	0	0	2	0	0	0	1
	α-Amylase	0	1	1	1	1	1	1
	Chitinase	1	0	0	0	0	1	1
	β-1,3-Glucanase	2	3	2	1	2	2	1
	Cellulase	0	3	2	3	3	4	2
	Protease	4	3	3	3	3	2	1

Only ≥40% similarity scores were considered. IAA, indole-3-acetic acid; ACC, 1-aminocyclopropane-1-carboxylate.

All the strains were predicted to be potentially able to fix nitrogen and produce nitric oxide, both useful features in agricultural practices (Ahmad et al., 2013), and to synthesize polyamines, as spermidine and putrescine, and the ACC deaminase, involved in lateral root development and plant growth enhancement under abiotic stress (Xie et al., 2014; Gupta and Pandey, 2019).

As expected, the genome of all the halophilic *Bacillus* strains contained multiple genes involved in antioxidant response, such as peroxidases, catalases, superoxide dismutase, and glutathione peroxidase (Hassan et al., 2020; Figure 3; Table 5). Other enzymes involved in abiotic stress responses were identified in the strains, as the osmoprotectants choline dehydrogenase, betaine-aldehyde dehydrogenase, and proline dehydrogenase (Table 5). The predicted production of osmotically active metabolites, as well as ROS scavenging enzymes, reflects the ability of the selected strains to survive in extreme environments, as salt-pans and to potentially alleviate abiotic stress in agricultural system.

Finally, all the isolates possessed in their genomes genes encoding for hydrolases involved in fungal cell-wall and starch degrading pathways, confirming the results obtained with the *in vitro* analysis, except for strain *B. frigoritolerans* RHFB whose genome did not carry α-amylase or cellulase genes.

### Antimicrobial Activity Screening

**Figure 4:**
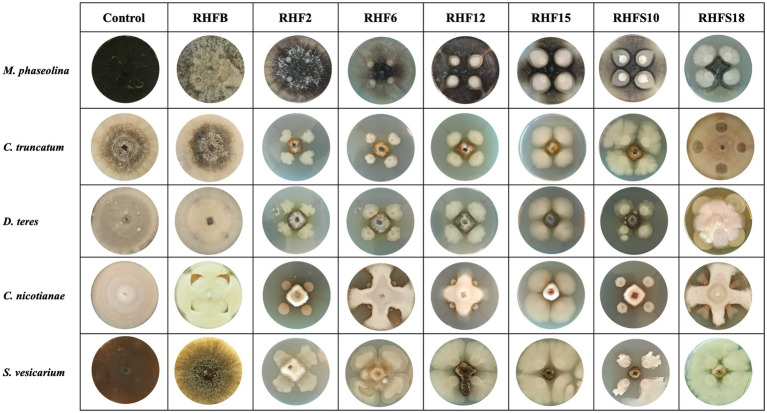
Representative photographs of dual culture assay for *in vitro* mycelial growth inhibition of fungal phytopathogens.

To verify the antagonistic potential that emerged from the genome-mining, the isolates were dually cultured with fungal and bacterial plant pathogens (see Table 1 for a list of the used phytopathogens). The results reveal that isolates inhibited plant pathogens growth on plates with different efficiency (Figure 4). Strains *B. subtilis* RHF2, *B. amyloliquefaciens* RHF6, and *Bacillus* sp. RHFS10 showed a broad inhibitory spectrum, being able to antagonize both phytopathogenic fungi and bacteria, while *B. halotolerans* RHF12 and *B. amyloliquefaciens* RHFS18 exhibited an antimicrobial activity limited to fungi. The highest antagonistic activity was observed for strain *Bacillus* sp. RHFS10, capable of inhibiting the growth of most of the test pathogens, confirming its biocontrol potential already observed by Castaldi et al. (2021). Unexpectedly, *B. frigoritolerans* RHFB exhibited no activity at all. Nevertheless, in the last decade, this species has been identified as a potential insect pathogenic bacterial species, with nematicidal activity (Selvakumar et al., 2011). The diversity observed in the antimicrobial activity against plant pathogens highlighted the phenotypic diversity of sand and rhizosphere isolated *Bacilli*, suggesting that in nature plant-associated bacteria may encounter different phytopathogens that may induce the acquisition of different antagonistic activity.

### Genome Mining for Biosynthetic Gene Clusters

**Figure 5:**
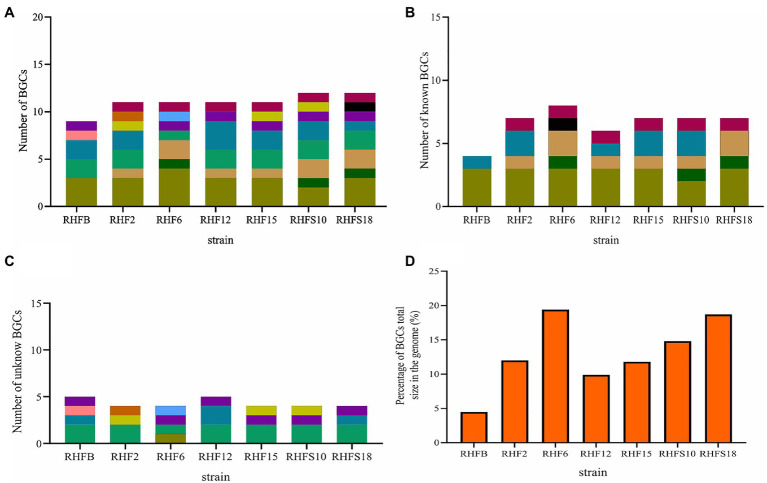
Number of biosynthetic gene clusters harbored by the strains and the percentage contribution of Biosynthetic Gene Clusters (BGCs) to the total genome size. **(A)** Total number of BGCs; **(B)** number of reported BGCs in the genomes; **(C)** number of unknown BGCs. BGCs that have different numbers of genes or show less than 70% protein identity to the reported ones were regarded as novel; and **(D)** the percentage contribution of BGCs to the genomes.

The biocontrol potential and the ability to enhance plant growth of PGPB are mostly attributed to their bioactive secondary metabolites. Proteins and metabolites released in the soil by PGPB, indeed, are implicated in root colonization, as well as in interactions with the plant immune response and the surrounding niche (Lugtenberg and Kamilova, 2009; Pieterse et al., 2014; Jamali et al., 2020). The strong antimicrobial activity of selected *Bacillus* strains is most likely due in part to the production of hydrolytic enzymes and siderophores observed in *in vitro* assays and confirmed by genome analysis (Tables 2 and 5). To better investigate this antagonistic activity, the biosynthetic potential of the halophilic PGPB was evaluated by using antiSMASH 6.0.0 to predict both characterized and unknown functioned secondary metabolites (Figure 5).

The bacterial isolates harbored BGCs coding for NRPSs, polyketide synthases (PKSs), post-translationally modified peptides (RiPPs), hybrid lipopeptides (NRPS-PKS; Figure 5A), and the majority of the BGCs are assigned to known products (Figure 5B; Supplementary Table S4). The unknown BGCs are type 3 polyketide synthase (T3PKS), RiPPs and terpenes (Figure 5C; Supplementary Table S4).

### Novel Non-ribosomal Peptide Synthetases and Bacteriocins

**Figure 6:**
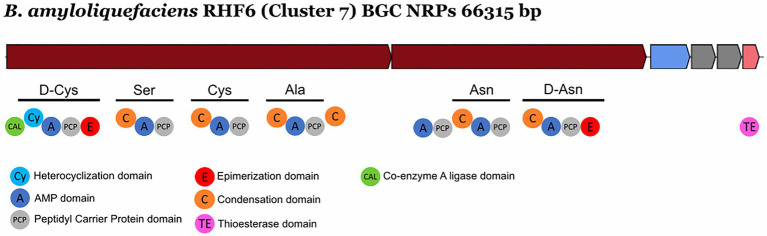
Novel NRP Biosynthetic gene Clusters identified from the isolate *Bacillus amyloliquefaciens* RHF6.

NRPs are modular enzymes that synthesize secondary metabolites, some of which are known to be involved in plant disease control (Ongena and Jacques, 2008). Several bioactive compounds produced by *Bacillus* strains fit in this category, such as surfactin or fengycin (Keswani et al., 2020), both of them exhibiting antimicrobial activity potentially exploited for biocontrol in agriculture. We have identified one novel BGC belonging to the class of the NRPs from *B. amyloliquefaciens* RHF6 (Figure 6). This cluster of 66.3 Kb has six genes encoding 25 domains, which include six condensation (C) domains, seven adenylation (A) domains, one coenzyme A ligase (CAL) domain, two epimerization (E) domains, one thioesterase (TE) domain, one heterocyclization (Cy) domain and seven peptidyl carrier protein (PCP) domains. Among them, 24 domains are essential components of this cluster, and catalyze the incorporation of seven amino acids into the final product exhibiting the following sequence: D-Cys-Ser-Cys-Ala-Asn-D-Asn. This cluster shows no similarity to any known BGCs reported in the antiSMASH database (Supplementary Table S4). The single heterocyclization (C) domain in the first module of the BGC, could form a thiazoline ring from a residue of cystine (Cys). Interestingly, many antimicrobial drugs expose a thiazoline ring (Desai et al., 2016). This allows us to speculate on the potential antimicrobial activity of the compound produced by this novel BGC.

**Figure 7:**
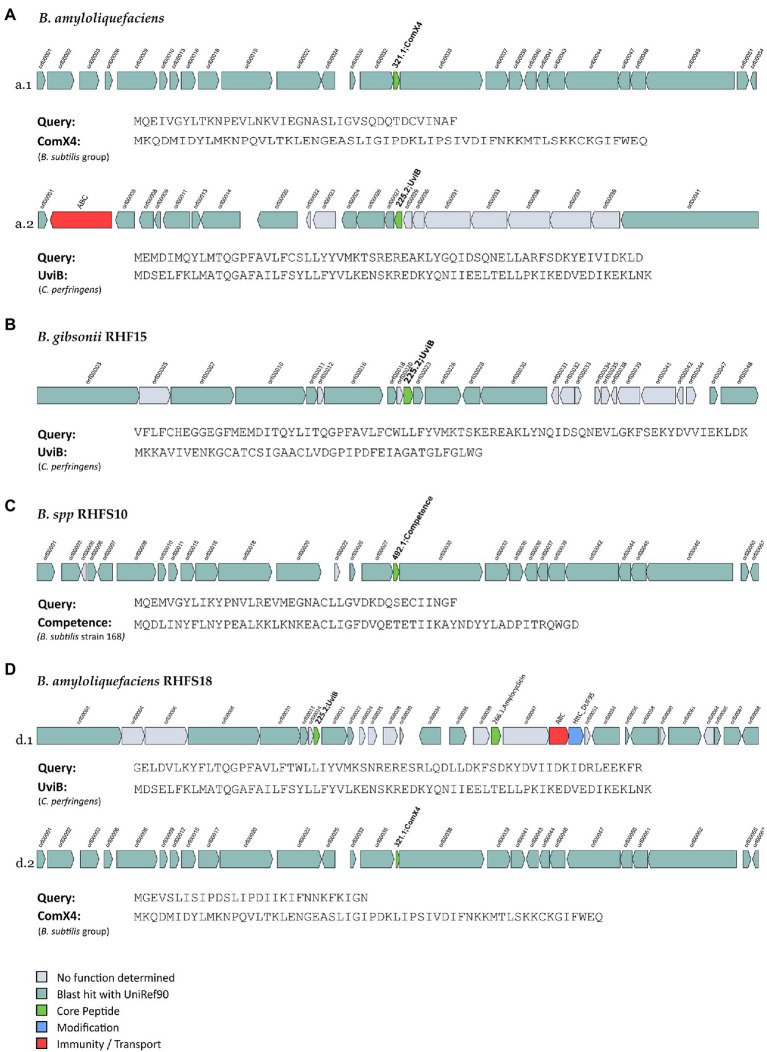
Novel bacteriocins identified from the isolated Bacillus strains (A: B. amyloliquefaciens, B: B. gibsonii RHF15, C: B. spp. RHFS10 and, D: B. amyloliquefaciens RHFS18). The BGCs identified from BAGEL4 analysis are shown and compared to the most similar available in BAGEL4 database.

The seven genomes were also mined for potential novel bacteriocins BGCs using BAGEL4. Bacteriocins are ribosomally synthesized antimicrobial peptides, generally active against bacteria closely related to producers (Cotter et al., 2013), and classified into three main classes: class I comprehends ribosomally produced and post-translationally modified peptides (RiPPs); class II unmodified peptides, and class III large antimicrobial peptides (Zhao and Kuipers, 2016). These molecules are directed against competitive microorganisms, and therefore generate a selective advantage for the producers. Generally, bacteriocins are highly specific against their target, although some might have a wider spectrum (Jack et al., 1995). The analysis made using BAGEL4, returned 15 regions of interest (in contrast with the antiSMASH analysis which revealed a higher number of bacteriocins, Supplementary Table S4), even though only six of them could be classified as novel bacteriocins, sharing ≤70% of similarity with known sequences from BAGEL4 database (Figure 7).

One orphan BGC of 27 genes is carried by both *B. amyloliquefaciens* RHF6 and RHFS18 strains (Figures 7a.1,d.1), although the core biosynthetic genes encode two different precursor peptides of 40 and 29 amino acids, respectively, sharing 41.03 and 57.14% of similarity with ComX4 from the *B. subtilis* group. In particular, ComX4 belongs to the ComX subclass of RiPPs according to the BAGEL4 database, and it is part of a major quorum-sensing system that regulates the development of genetic competence (Okada et al., 2005) and the production of surfactins (Caulier et al., 2019). *Bacillus amyloliquefaciens* RHF6 also harbors a BGC of 23 genes (Figure 7A-a.2), with the core biosynthetic gene encoding a 63-amino acids precursor peptide, showing a similarity of 36.51% compared to UviB, a class II bacteriocin first identified in the mobilizable plasmid pIP404, from *C. perfringens*, known to be bacteriocinogenic (Garnier and Cole, 1988). Interestingly, two different BGCs containing the same gene encoding for a putative UviB-like bacteriocin, were found in strains *B. gibsonii* RHF15 (Figure 7B) and *B. amyloliquefaciens* RHFS18 (7D-d.1). Their precursor peptides share 42.1 and 33.4% similarity with UviB.

Finally, *Bacillus* sp. RHFS10 carries an orphan 28 genes BGC with a core biosynthetic gene encoding a 40-amino acids peptide sharing 35% of similarity with the competence pheromone of *B. subtilis 168*, a RiPP belonging to class I bacteriocins. *Bacillus* species are known to synthesize many well-studied bacteriocins, such as subtilin, ericin, paenibacillin, subtilosin, thuricin, and coagulin (Abriouel et al., 2011). Anyway, it is impossible to predict if the six compounds produced by strains *B. amyloliquefaciens* RHF6, and RHFS18, *B. gibsonii* RHF15 and *Bacillus* sp. RHFS10 actually have antimicrobial properties from genome sequence data only. Despite this, the antagonistic activity exerted by RHF6, RHF 15, RHFS10, and RHFS18 strains observed previously in *in vitro* assays (Table 6) could be associated with these potential compounds. This will need to be validated by further experiments.

**Table 6:** Antimicrobial activity of the seven selected strains against phytopathogenic fungi and bacteria.

Pathogen types	Species	RHFB	RHF2	RHF6	RHF12	RHF15	RHFS10	RHFS18
Fungi	*M. phaseolina*	−	−	−	+	++	+++	+++
	*C. truncatum*	−	−	+++	+++	+++	+++	+++
	*D. teres*	−	−	+++	+++	+++	+++	+++
	*C. nicotianae*	−	+++	++	++	+++	+++	++
	*S. vesicarium*	−	++	+++	++	+++	+++	−
Bacteria	*P. tolaasii*	−	−	+	−	−	+	−
	*P. syringae pv tabaci*	−	++	++	−	−	+	−
	*P. syringae pv panici*	−	++	++	−	−	+	−
	*P. cariophilly*	−	−	−	−	+	+	−
	*P. syringae pv syringae*	−	+	+	−	−	++	−
	*P. syringae pv japonica*	−	++	++	−	−	+	−
	*P. syringae pv papulans*	−	−	−	−	−	−	++

No inhibition (−), inhibitory zone <5mm (+), inhibitory zone 5mm (++), and inhibitory zone >5mm (+++).

## Conclusion

In a historic moment in which the increasing population coupled with land degradation aggravates crop production, the use of plant growth promoting bacteria to ensure agricultural productivity has a huge impact on our society. These soil microorganisms enhance plant performance and represent an eco-friendly alternative to chemical fertilizers and pesticides (Hashem et al., 2019). When applied directly to the soil, PGPB enhance plant growth by different action mechanisms such as the production of different phytohormones, accelerating the mineralization of organic matter and improving the bioavailability of the nutrients, and protecting plants from pests’ damages. The beneficial activity exerted by PGPB is in part mediated by a broad spectrum of secondary metabolites and enzymes. For example, polyamines, such as spermidine, play important physiological and protective roles in plants, resulting in an increase in biomass, altered root architecture, and elevated photosynthetic capacity. Until recently, these key metabolites were uncovered only by systematic investigation or by serendipity, often understating the PGPB potentiality during their screening. Many genes involved in PGB activity, in fact, could be silent under standard laboratory conditions, due to the absence of appropriate natural triggers or stress signals. More recently, the onset of the genomic era has facilitated the discovery of these ecologically important metabolites and novel strategies became available for PGPR characterization.

For example, genome mining allows to look over the whole genome of a PGPB strain and highlights genes encoding beneficial enzymes, involved in the enhancement of plant nutritional uptake or modulation of hormone levels, as well as for antimicrobial-encoding BGCs.

In this work, we have isolated soil halophilic *Bacilli* and performed their screening for PGP traits by using standard laboratory procedures and whole-genome analysis. *Bacilli* represent a significant fraction of the soil microbial community and some species are categorized as PGPB (Cazorla et al., 2007). They are also able to produce endospores, which besides enduring harsh environmental conditions fatal for other cell forms (Petrillo et al., 2020), permit easy formulation and storage of commercial PGPB-based products. In addition, salt-tolerant PGPB can easily withstand several abiotic stresses and ameliorate plant growth in degraded soil.

Seven *Bacillus* strains have been selected for *in vitro* PGP traits and identified at the species level by genome analysis. Based on genome mining, not only have we confirmed the beneficial activities PGP found by *in vitro* analysis, identifying the involved genes but also we have highlighted their strong potentiality by the discovery of novel biosynthesis gene clusters. Our results demonstrated that the genomic analyses, as genome mining, allow a full investigation of PGPB biosynthetic capacity for secondary metabolites and proteins and represent useful tools in the characterization of plant beneficial bacteria. Nevertheless, the divergences observed between the predicted biocontrol functions by found gene clusters and the results obtained by *in vitro* analysis, highlight the need of combining laboratory-assays and genome-mining in identification of new PGPB for future applications.

## Data Availability Statement

The datasets presented in this study can be found in online repositories. The names of the repository/repositories and accession number(s) can be found in the article/Supplementary Material.

## Author Contributions

RI: conceptualization, supervision, project administration, and funding acquisition. SC and CP: methodology. SC, CP, ML, and MS: validation and formal analysis. SC, CP, and DG: investigation. SC, CP, MS, AC, and RI: data curation. RI, SC, CP, and DG: writing original draft preparation. All authors have read and agreed to the published version of the manuscript.

## Conflict of Interest

The authors declare that the research was conducted in the absence of any commercial or financial relationships that could be construed as a potential conflict of interest.

## Publisher’s Note

All claims expressed in this article are solely those of the authors and do not necessarily represent those of their affiliated organizations, or those of the publisher, the editors and the reviewers. Any product that may be evaluated in this article, or claim that may be made by its manufacturer, is not guaranteed or endorsed by the publisher.

## Footnotes

^1^ https://gtdb.ecogenomic.org

## References

[ref1] Abriouel H. Franz C. M. A. P. Ben Omar N. Gálvez A. (2011). Diversity and applications of *Bacillus* bacteriocins. FEMS Microbiol. Rev. 35, 201–232. doi: 10.1111/j.1574-6976.2010.00244.x, PMID: 20695901

[ref2] Agrawal A. A. (2001). Phenotypic plasticity in the interactions and evolution of species. Science 294, 321–326. doi: 10.1126/science.1060701, PMID: 11598291

[ref3] Ahmad M. Zahir Z. A. Khalid M. Nazli F. Arshad M. (2013). Efficacy of Rhizobium and *Pseudomonas* strains to improve physiology, ionic balance and quality of mung bean under salt-affected conditions on farmer’s fields. Plant Physiol. Biochem. 63, 170–176. doi: 10.1016/j.plaphy.2012.11.024, PMID: 23262185

[ref300] Adler J. (1966). Chemotaxis in Bacteria. Science. 153, 708–716. doi: 10.1126/science.153.3737.708, PMID: 4957395

[ref4] Amaya-Gómez C. V. Porcel M. Mesa-Garriga L. Gómez-Álvarez M. I. (2020). A framework for the selection of plant growth-promoting rhizobacteria based on bacterial competence mechanisms. Appl. Environ. Microbiol. 86, e00760–e00820. doi: 10.1128/AEM.00760-20, PMID: 32358015 PMC7357491

[ref5] Anwar U. B. Zwar I. P. de Souza A. O. (2020). “Chapter 12: Biomolecules produced by extremophiles microorganisms and recent discoveries,” in New and Future Developments in Microbial Biotechnology and Bioengineering. ed. Rodrigues A. G. (Elsevier), 247–270.

[ref6] Babalola O. O. (2010). Beneficial bacteria of agricultural importance. Biotechnol. Lett. 32, 1559–1570. doi: 10.1007/s10529-010-0347-0, PMID: 20635120

[ref7] Blin K. Shaw S. Steinke K. Villebro R. Ziemert N. Lee S. Y.. (2019). antiSMASH 5.0: updates to the secondary metabolite genome mining pipeline. Nucleic Acids Res. 47, W81–W87. doi: 10.1093/nar/gkz310, PMID: 31032519 PMC6602434

[ref8] Bolger A. M. Lohse M. Usadel B. (2014). Trimmomatic: a flexible trimmer for Illumina sequence data. Bioinformatics 30, 2114–2120. doi: 10.1093/bioinformatics/btu170, PMID: 24695404 PMC4103590

[ref9] Bunsangiam S. Sakpuntoon V. Srisuk N. Ohashi T. Fujiyama K. Limtong S. (2019). Biosynthetic pathway of indole-3-acetic acid in basidiomycetous yeast rhodosporidiobolus fluvialis. Mycobiology 47, 292–300. doi: 10.1080/12298093.2019.1638672, PMID: 31565465 PMC6758620

[ref10] Cangiano G. Mazzone A. Baccigalupi L. Isticato R. Eichenberger P. De Felice M.. (2010). Direct and indirect control of late sporulation genes by GerR of *Bacillus subtilis*. J. Bacteriol. 192, 3406–3413. doi: 10.1128/JB.00329-10, PMID: 20435725 PMC2897654

[ref11] Cangiano G. Sirec T. Panarella C. Isticato R. Baccigalupi L. De Felice M.. (2014). The sps gene products affect the germination, hydrophobicity, and protein adsorption of *Bacillus subtilis* spores. Appl. Environ. Microbiol. 80, 7293–7302. doi: 10.1128/AEM.02893-14, PMID: 25239894 PMC4249184

[ref12] Castaldi S. Petrillo C. Donadio G. Piaz F. D. Cimmino A. Masi M.. (2021). Plant growth promotion function of *Bacillus* sp. strains isolated from salt-pan rhizosphere and their biocontrol potential against macrophomina phaseolina. Int. J. Mol. Sci. 22:3324. doi: 10.3390/ijms22073324, PMID: 33805133 PMC8036593

[ref13] Caulier S. Nannan C. Gillis A. Licciardi F. Bragard C. Mahillon J. (2019). Overview of the antimicrobial compounds produced by members of the *Bacillus subtilis* group. Front. Microbiol. 10:302. doi: 10.3389/fmicb.2019.00302, PMID: 30873135 PMC6401651

[ref14] Cazorla F. M. Romero D. Pérez-García A. Lugtenberg B. J. J. de Vicente A. Bloemberg G. (2007). Isolation and characterization of antagonistic *Bacillus subtilis* strains from the avocado rhizoplane displaying biocontrol activity. J. Appl. Microbiol. 103, 1950–1959. doi: 10.1111/j.1365-2672.2007.03433.x, PMID: 17953605

[ref15] Celandroni F. Vecchione A. Cara A. Mazzantini D. Lupetti A. Ghelardi E. (2019). Identification of *Bacillus* species: implication on the quality of probiotic formulations. PLoS One 14:e0217021. doi: 10.1371/journal.pone.0217021, PMID: 31107885 PMC6527297

[ref16] Cotter P. D. Ross R. P. Hill C. (2013). Bacteriocins: a viable alternative to antibiotics? Nat. Rev. Microbiol. 11, 95–105. doi: 10.1038/nrmicro2937, PMID: 23268227

[ref1400] Corrado I. Petrillo C. Isticato R. Casillo A. Corsaro M. M. Sannia G.. (2021). The power of two: An artificial microbial consortium for the conversion of inulin into Polyhydroxyalkanoates. Int. J. Biol. Macromol. 189, 494–502. doi: 10.1016/.ijbiomac.2021.08.123, PMID: 34428488

[ref17] Damodaran T. Sah V. Rai R. B. Sharma D. K. Mishra V. K. Jha S. K.. (2013). Isolation of salt tolerant endophytic and rhizospheric bacteria by natural selection and screening for promising plant growth-promoting rhizobacteria (PGPR) and growth vigour in tomato under sodic environment. Afr. J. Microbiol. Res. 7, 5082–5089. doi: 10.5897/AJMR2013.6003

[ref18] Desai N. C. Makwana A. H. Rajpara K. M. (2016). Synthesis and study of 1,3,5-triazine based thiazole derivatives as antimicrobial agents. J. Saudi Chem. Soc. 20, S334–S341. doi: 10.1016/j.jscs.2012.12.004

[ref400] Eren A. M. Kiefl E. Shaiber A. Veseli I. Miller S. E. Schechter M. S.. (2021). Community-led, integrated, reproducible multi-omics with anvi’o. Microbiol. 6, 3–6. doi: 10.1038/s41564-020-00834-3, PMID: 33349678 PMC8116326

[ref19] Etesami H. Mirsyed Hosseini H. Alikhani H. A. Mohammadi L. (2014). Bacterial biosynthesis of 1-aminocyclopropane-1-carboxylate (ACC) deaminase and indole-3-acetic acid (IAA) as endophytic preferential selection traits by rice plant seedlings. J. Plant Growth Regul. 33, 654–670. doi: 10.1007/s00344-014-9415-3, PMID: 25320466

[ref20] Garnier T. Cole S. T. (1988). Complete nucleotide sequence and genetic organization of the bacteriocinogenic plasmid, pIP404, from *Clostridium perfringens*. Plasmid 19, 134–150. doi: 10.1016/0147-619X(88)90052-2, PMID: 2901768

[ref21] Giglio R. Fani R. Isticato R. De Felice M. Ricca E. Baccigalupi L. (2011). Organization and evolution of the cotG and cotH genes of *Bacillus subtilis*. J. Bacteriol. 193, 6664–6673. doi: 10.1128/JB.06121-11, PMID: 21984783 PMC3232876

[ref22] Glick B. R. Cheng Z. Czarny J. Duan J. (2007). Promotion of plant growth by ACC deaminase-producing soil bacteria. Eur. J. Plant Pathol. 119, 329–339. doi: 10.1007/s10658-007-9162-4

[ref23] Goberna M. García C. Verdú M. (2014). A role for biotic filtering in driving phylogenetic clustering in soil bacterial communities. Glob. Ecol. Biogeogr. 23, 1346–1355. doi: 10.1111/geb.12227

[ref24] Gordon S. A. Weber R. P. (1951). Colorimetric estimation of indoleacetic acid. Plant Physiol. 26, 192–195. doi: 10.1104/pp.26.1.192, PMID: 16654351 PMC437633

[ref25] Gupta S. Pandey S. (2019). ACC deaminase producing bacteria with multifarious plant growth promoting traits alleviates salinity stress in french bean (*Phaseolus vulgaris*) plants. Front. Microbiol. 10:1506. doi: 10.3389/fmicb.2019.01506, PMID: 31338077 PMC6629829

[ref26] Hashem A. Tabassum B. Fathi Abd Allah E. (2019). *Bacillus subtilis*: a plant-growth promoting rhizobacterium that also impacts biotic stress. Saudi J. Biol. Sci. 26, 1291–1297. doi: 10.1016/j.sjbs.2019.05.004, PMID: 31516360 PMC6734152

[ref27] Hassan A. H. A. Alkhalifah D. H. M. Al Yousef S. A. Beemster G. T. S. Mousa A. S. M. Hozzein W. N.. (2020). Salinity stress enhances the antioxidant capacity of *Bacillus* and *Planococcus* species isolated from saline lake environment. Front. Microbiol. 11:561816. doi: 10.3389/fmicb.2020.561816, PMID: 33042068 PMC7521018

[ref28] Hortal J. Carrascal L. Triantis K. Thébault E. Meiri S. (2013). Species richness can decrease with altitude but not with habitat diversity. Proc. Natl. Acad. Sci. U. S. A. 110, E2149–E2150. doi: 10.1073/pnas.1301663110, PMID: 23661060 PMC3683747

[ref29] Jack R. W. Tagg J. R. Ray B. (1995). Bacteriocins of gram-positive bacteria. Microbiol. Rev. 59, 171–200. doi: 10.1128/mr.59.2.171-200.1995, PMID: 7603408 PMC239359

[ref30] Jadhav H. Shaikh S. Sayyed R. (2017). “Role of hydrolytic enzymes of rhizoflora in biocontrol of fungal phytopathogens: an overview,” in Rhizotrophs: Plant Growth Promotion to Bioremediation. ed. Mehnaz S. (Singapore: Springer Singapore) 183–203.

[ref31] Jain C. Rodriguez-R L. M. Phillippy A. M. Konstantinidis K. T. Aluru S. (2018). High throughput ANI analysis of 90K prokaryotic genomes reveals clear species boundaries. Nat. Commun. 9:5114. doi: 10.1038/s41467-018-07641-9, PMID: 30504855 PMC6269478

[ref32] Jamali H. Sharma A. Roohi Srivastava A. K. (2020). Biocontrol potential of *Bacillus subtilis* RH5 against sheath blight of rice caused by Rhizoctonia solani. J. Basic Microbiol. 60, 268–280. doi: 10.1002/jobm.201900347, PMID: 31851769

[ref33] Keswani C. Singh H. B. García-Estrada C. Caradus J. He Y.-W. Mezaache-Aichour S.. (2020). Antimicrobial secondary metabolites from agriculturally important bacteria as next-generation pesticides. Appl. Microbiol. Biotechnol. 104, 1013–1034. doi: 10.1007/s00253-019-10300-8, PMID: 31858191

[ref34] Kumar A. Prakash A. Johri B. (2011). “Bacillus as PGPR in crop ecosystem,” in Bacteria in Agrobiology: Crop Ecosystems (Springer Berlin Heidelberg), 37–59.

[ref35] Li Z. Chakraborty P. de Vries R. H. Song C. Zhao X. Roelfes G.. (2020). Characterization of two relacidines belonging to a novel class of circular lipopeptides that act against gram-negative bacterial pathogens. Environ. Microbiol. 22, 5125–5136. doi: 10.1111/1462-2920.15145, PMID: 32608161 PMC7818431

[ref36] Lugtenberg B. Kamilova F. (2009). Plant-growth-promoting rhizobacteria. Annu. Rev. Microbiol. 63, 541–556. doi: 10.1146/annurev.micro.62.081307.162918, PMID: 19575558

[ref37] O’Toole G. A. (2011). Microtiter dish biofilm formation assay. J. Vis. Exp. 30:2437. doi: 10.3791/2437, PMID: 21307833 PMC3182663

[ref38] Okada M. Sato I. Cho S. J. Iwata H. Nishio T. Dubnau D.. (2005). Structure of the *Bacillus subtilis* quorum-sensing peptide pheromone ComX. Nat. Chem. Biol. 1, 23–24. doi: 10.1038/nchembio709, PMID: 16407988

[ref39] Ongena M. Jacques P. (2008). Bacillus lipopeptides: versatile weapons for plant disease biocontrol. Trends Microbiol. 16, 115–125. doi: 10.1016/j.tim.2007.12.009, PMID: 18289856

[ref40] Pal K. K. McSpadden Gardener B. (2006). Biological control of plant pathogens. Plant Health Instr. 2, 1117–1142. doi: 10.1094/PHI-A-2006-1117-02

[ref41] Pérez-Miranda S. Cabirol N. George-Téllez R. Zamudio-Rivera L. S. Fernández F. J. (2007). O-CAS, a fast and universal method for siderophore detection. J. Microbiol. Methods 70, 127–131. doi: 10.1016/j.mimet.2007.03.023, PMID: 17507108

[ref42] Pertot I. Caffi T. Rossi V. Mugnai L. Hoffmann C. Grando M. S.. (2017). A critical review of plant protection tools for reducing pesticide use on grapevine and new perspectives for the implementation of IPM in viticulture. Crop Prot. 97, 70–84. doi: 10.1016/j.cropro.2016.11.025

[ref43] Pesce G. Rusciano G. Sasso A. Isticato R. Sirec T. Ricca E. (2014). Surface charge and hydrodynamic coefficient measurements of *Bacillus subtilis* spore by optical tweezers. Colloids Surf. B Biointerfaces 116, 568–575. doi: 10.1016/j.colsurfb.2014.01.039, PMID: 24583259

[ref44] Petrillo C. Castaldi S. Lanzilli M. Saggese A. Donadio G. Baccigalupi L.. (2020). The temperature of growth and sporulation modulates the efficiency of spore-display in *Bacillus subtilis*. Microb. Cell Factories 19:185. doi: 10.1186/s12934-020-01446-6, PMID: 33004043 PMC7528486

[ref45] Pieterse C. M. J. Zamioudis C. Berendsen R. L. Weller D. M. Van Wees S. C. M. Bakker P. A. H. M. (2014). Induced systemic resistance by beneficial microbes. Annu. Rev. Phytopathol. 52, 347–375. doi: 10.1146/annurev-phyto-082712-102340, PMID: 24906124

[ref46] Pikovskaya R. I. (1948). Mobilization of phosphorus in soil in connection with the vital activity of some microbial species. Mikrobiologiya 17, 362–370.

[ref47] Reddy K. R. N. Reddy C. S. Muralidharan K. (2009). Potential of botanical and biocontrol agents on growth and aflatoxin production by Aspergillus flavus infecting rice grains. Food Control 20, 173–178. doi: 10.1016/j.foodcont.2008.03.009

[ref48] Rodríguez-Echeverría S. Lozano Y. M. Bardgett R. D. (2016). Influence of soil microbiota in nurse plant systems. Funct. Ecol. 30, 30–40. doi: 10.1111/1365-2435.12594

[ref49] Rooney A. P. Price N. P. J. Ehrhardt C. Swezey J. L. Bannan J. D. (2009). Phylogeny and molecular taxonomy of the *Bacillus subtilis* species complex and description of *Bacillus subtilis* subsp. inaquosorum subsp. nov. Int. J. Syst. Evol. Microbiol. 59, 2429–2436. doi: 10.1099/ijs.0.009126-0, PMID: 19622642

[ref50] Schiraldi C. De Rosa M. (2016). “Mesophilic organisms,” in Encyclopedia of Membranes. eds. Drioli E. Giorno L. (Berlin, Heidelberg: Springer Berlin Heidelberg), 1–2.

[ref51] Schoebitz M. Ceballos C. Ciampi L. (2013). Effect of immobilized phosphate solubilizing bacteria on wheat growth and phosphate uptake. J. Soil Sci. Plant Nutr. 13, 1–10. doi: 10.4067/S0718-95162013005000001

[ref52] Seemann T. (2014). Prokka: rapid prokaryotic genome annotation. Bioinformatics 30, 2068–2069. doi: 10.1093/bioinformatics/btu153 24642063

[ref53] Selvakumar G. Sushil S. N. Stanley J. Mohan M. Deol A. Rai D.. (2011). Brevibacterium frigoritolerans a novel entomopathogen of Anomala dimidiata and Holotrichia longipennis (Scarabaeidae: Coleoptera). Biocontrol Sci. Tech. 21, 821–827. doi: 10.1080/09583157.2011.586021

[ref54] Shultana R. Kee Zuan A. T. Yusop M. R. Saud H. M. (2020). Characterization of salt-tolerant plant growth-promoting rhizobacteria and the effect on growth and yield of saline-affected rice. PLoS One 15:e0238537. doi: 10.1371/journal.pone.0238537, PMID: 32886707 PMC7473536

[ref55] Sitbon F. Astot C. Edlund A. Crozier A. Sandberg G. (2000). The relative importance of tryptophan-dependent and tryptophan-independent biosynthesis of indole-3-acetic acid in tobacco during vegetative growth. Planta 211, 715–721. doi: 10.1007/s004250000338, PMID: 11089685

[ref56] van Heel A. J. de Jong A. Song C. Viel J. H. Kok J. Kuipers O. P. (2018). BAGEL4: a user-friendly web server to thoroughly mine RiPPs and bacteriocins. Nucleic Acids Res. 46, W278–W281. doi: 10.1093/nar/gky383, PMID: 29788290 PMC6030817

[ref57] Ventosa A. Nieto J. J. Oren A. (1998). Biology of moderately halophilic aerobic bacteria. Microbiol. Mol. Biol. Rev. 62, 504–544. doi: 10.1128/MMBR.62.2.504-544.1998, PMID: 9618450 PMC98923

[ref58] Xie S.-S. Wu H.-J. Zang H.-Y. Wu L.-M. Zhu Q.-Q. Gao X.-W. (2014). Plant growth promotion by spermidine-producing *Bacillus subtilis* OKB105. Mol. Plant-Microbe Interact. 27, 655–663. doi: 10.1094/MPMI-01-14-0010-R, PMID: 24678831

[ref59] Xu S. J. Kim B. S. (2014). Biocontrol of fusarium crown and root rot and promotion of growth of tomato by paenibacillus strains isolated from soil. Mycobiology 42, 158–166. doi: 10.5941/MYCO.2014.42.2.158 25071385 PMC4112232

[ref60] Yoon S.-H. Ha S.-M. Lim J. Kwon S. Chun J. (2017). A large-scale evaluation of algorithms to calculate average nucleotide identity. Antonie Van Leeuwenhoek 110, 1281–1286. doi: 10.1007/s10482-017-0844-4, PMID: 28204908

[ref61] Zhao X. Kuipers O. P. (2016). Identification and classification of known and putative antimicrobial compounds produced by a wide variety of Bacillales species. BMC Genomics 17:882. doi: 10.1186/s12864-016-3224-y, PMID: 27821051 PMC5100339

