# Supplementary material for: Genomic and Physiological Characterization of Bacilli Isolated From Salt-Pans With Plant Growth Promoting Features
Source: Front Microbiol. 2021 Sep 13;12:715678. doi: 10.3389/fmicb.2021.715678 (PMC8475271; doi:10.3389/fmicb.2021.715678)
Supplement: Supplementary file 1 [file Data_Sheet_1.docx]

Supplementary Material

**Table S1.** The minimum information about genome sequences (MIGS).

| **MIGS ID** | **Property** | **Term** |
| --- | --- | --- |
| MIGS-31 | Finishing quality | High-Quality Draft |
| MIGS-28 | Libraries used | Illumina MiSeq Reagent Kit v2 2x250bp paired-end reads |
| MIGS-29 | Sequencing platforms | Illumina MiSeq |
| MIGS-31.2 | Fold coverage | 30x |
| MIGS-30 | Assemblers | SPAdes version 3.7 |
| MIGS-32 | Gene calling method | Microbial Genome Annotation Pipeline (MiGAP) |
|  | Genbank ID | SAMN17389615 (RHFB), SAMN17389609 (RHF2), SAMN17389610 (RHF6), SAMN17389612(RHF12), SAMN17389613 (RHF15), SAMN17389611 (RHFS10), SAMN17389614 (RHFS18) |
|  | Genbank Date of Release | 03/09/2021 |
|  | BIOPROJECT | PRJNA693507 |
|  | Project relevance | Industrial |

**Table S2.** Preliminary characterization of spore-forming bacteria isolated from salt-pans.

| **Strain** | **Colony colour** | **Colony morphology** | ***Anaerobic growth** | **pH range** | **Temperature range (°C)** | **Salinity NaCl (%)** |
| --- | --- | --- | --- | --- | --- | --- |
| **RHF1** | Creamy-white | Circular | ++ | 4-12 | 15-50 | 0.8-10 |
| **RHF2** | Creamy-white | Flat | +++ | 6-10 | 15-50 | 0.8-10 |
| **RHF3** | Creamy-white | Irregular | ++ | 4-12 | 15-50 | 0.8-10 |
| **RHF4** | Milky-white | Undulate | + | 4-12 | 15-50 | 0.8-10 |
| **RHF5** | Creamy-white | Circular | ++ | 4-12 | 15-60 | 0.8-10 |
| **RHF6** | Creamy-white | Flat | +++ | 4-10 | 15-50 | 0.8-13 |
| **RHF7** | Milky-white | Circular | +++ | 4-12 | 15-50 | 0.8-10 |
| **RHF8** | Creamy-white | Circular | + | 4-10 | 15-50 | 0.8-8 |
| **RHF9** | Creamy-white | Irregular | + | 4-10 | 15-50 | 0.8-10 |
| **RHF10** | Creamy-white | Circular | + | 6-12 | 15-50 | 0.8-10 |
| **RHF11** | Brown | Rhizoid | ++ | 6-12 | 15-50 | 0.8-10 |
| **RHF12** | Creamy-white | Wrinkled | +++ | 4-10 | 15-50 | 0.8-10 |
| **RHF13** | Orange | Circular | ++ | 4-12 | 15-50 | 0.8-10 |
| **RHF14** | Creamy-white | Circular | ++ | 4-10 | 15-50 | 0.8-10 |
| **RHF15** | Creamy-white | Wrinkled | +++ | 4-10 | 15-50 | 0.8-10 |
| **RHF16** | Creamy-white | Undulate | ++ | 4-10 | 15-50 | 0.8-8 |
| **RHF17** | Creamy-white | Rhizoid | ++ | 6-12 | 15-50 | 0.8-10 |
| **RHFB** | Brown | Irregular | + | 6-10 | 15-37 | 0.8-5 |
| **RHFE** | Creamy-white | Irregular | ++ | 6-12 | 15-50 | 0.8-10 |
| **RHFL** | Yellow | Translucent | ++ | 6-12 | 15-50 | 0.8-10 |

*Anaerobic growth: +: low growth; ++: moderate growth; +++: high growth

**Table S3.** Pairwise average nucleotide identities (ANI) between the isolated strains and the closest relatives identified in the polyphasic analysis.

|  | **RHFB** | **RHF2** | **RHF6** | **RHF12** | **RHF15** | **RHFS10** | **RHFS18** |
| --- | --- | --- | --- | --- | --- | --- | --- |
| *B. subtilis 168* | 68.21 | **99.96** | 77.28 | 87.34 | 98.79 | 91.84 | 76.88 |
| *B. gibsonii* | 68.47 | 98.83 | 77.09 | 87.3 | **99.6** | 91.76 | 77.01 |
| *B. amyloliquefaciens* | 67.94 | 77.1 | **99.26** | 77.17 | 76.83 | 77.13 | **98.36** |
| *B. velezensis* | 68.23 | 77.25 | 99.15 | 77.21 | 77.12 | 77.12 | 98.35 |
| *B. halotolerans* | 68.38 | 87.45 | 77.42 | **98.04** | 87.38 | 87.79 | 77.02 |
| *B. frigoritolerans* | **96.95** | 68.46 | 67.61 | 68.47 | 68.56 | 68.17 | 67.94 |
| *B. vallismortis* | 68.25 | 91.01 | 77.16 | 87.29 | 90.87 | **93.48** | 77.33 |

**Table S4.** Identified PGPR gene clusters and similarity (amino acid identity) to the closest organism (when available).

| **Strain and gene cluster** | **Length (bp)** | **Type** | **Compound** | **Similarity (%)** | **Organism** |
| --- | --- | --- | --- | --- | --- |
| **RHFB** |  |  |  |  |  |
| Cluster 1 | 24169 | NRPS | fengycin | 46 | *B. velezensis FZB42* |
| Cluster 2 | 23535 | RiPP (LAP) | unknown |  |  |
| Cluster 3 | 20818 | terpene | unknown |  |  |
| Cluster 4 | 16393 | RiPP | paeninodin | 100 | *Paenibacillus dendritiformis C454* |
| Cluster 5 | 21895 | terpene | unknown |  |  |
| Cluster 6 | 15513 | siderophore | unknown |  |  |
| Cluster 7 | 41088 | T3PKS | unknown |  |  |
| Cluster 8 | 49726 | NRPS | koraminine | 87 | *B. sp. NK2003* |
| Cluster 9 | 43445 | NRPS | bacillibactin | 53 | *B. subtilis subsp. subtilis str. 168* |
| **RHF2** |  |  |  |  |  |
| Cluster 1 | 20518 | terpene | unknown |  |  |
| Cluster 2 | 114759 | Hybrid PKS/NRPS | bacillaene | 100 | *B. velezensis FZB42* |
| Cluster 3 | 72650 | NRPS | fengycin | 100 | *B. velezensis FZB42* |
| Cluster 4 | 41097 | terpene | unknown |  |  |
| Cluster 5 | 20746 | CDPS | unknown |  |  |
| Cluster 6 | 49741 | NRPS | bacillibactin | 100 | *B. subtilis subsp. subtilis str. 168* |
| Cluster 7 | 65391 | NRPS | surfactin | 82 | *B. velezensis FZB42* |
| Cluster 8 | 41418 | Other | bacilysin | 100 | *B. velezensis FZB42* |
| Cluster 9 | 21611 | RiPP (Thiopeptide) | subtilosin A | 100 | *B. subtilis subsp. spizizenii ATCC 6633* |
| Cluster 10 | 22953 | RiPP (Head-to-tailcyclized peptide) | sporulation killing factor | 100 | *B. subtilis subsp. subtilis str. 168* |
| Cluster 11 | 11461 | RRE-containing | unknown |  |  |
| **RHF6** |  |  |  |  |  |
| Cluster 1 | 105763 | Hybrid PKS/NRPS | difficidin | 100 | *B. velezensis FZB42* |
| Cluster 2 | 40094 | T3PKS | unknown |  |  |
| Cluster 3 | 119121 | NRPS | fengycin | 93 | *B. velezensis FZB42* |
| Cluster 4 | 109377 | Hybrid PKS/NRPS | bacillaene | 100 | *B. velezensis FZB42* |
| Cluster 5 | 88230 | PKS | macrolactin H | 100 | *B. velezensis FZB42* |
| Cluster 6 | 20740 | terpene | unknown |  |  |
| Cluster 7 | 66315 | NRPS | unknown |  |  |
| Cluster 8 | 51793 | NRPS | bacillibactin | 100 | *B. subtilis subsp. subtilis str. 168* |
| Cluster 9 | 41418 | Other | bacilysin | 100 | *B. velezensis FZB42* |
| Cluster 10 | 65407 | NRPS | surfactin | 82 | *B. velezensis FZB42* |
| Cluster 11 | 41244 | Saccharide | butirosin A/ butirosin B | 7 | *B. circulans* |
| Cluster 12 | 40884 | Phosphonate | unknown |  |  |
| **RHF12** |  |  |  |  |  |
| Cluster 1 | 60718 | NRPS | surfactin | 82 | *B. velezensis FZB42* |
| Cluster 2 | 41097 | T3PKS | unknown |  |  |
| Cluster 3 | 21612 | RiPP (Thiopeptide) | subtilosin A | 100 | *B. subtilis subsp. spizizenii ATCC 6633* |
| Cluster 4 | 41418 | Other | bacilysin | 100 | *B. velezensis FZB42* |
| Cluster 5 | 95323 | Hybrid PKS/NRPS | bacillaene | 92 | *B. velezensis FZB42* |
| Cluster 6 | 49738 | NRPS | bacillibactin | 100 | *B. subtilis subsp. subtilis str. 168* |
| Cluster 7 | 62913 | NRPS | fengycin | 93 | *B. velezensis FZB42* |
| Cluster 8 | 14447 | terpene | unknown |  |  |
| Cluster 9 | 11709 | terpene | unknown |  |  |
| Cluster 10 | 5195 | lanthipeptide-classIII | unknown |  |  |
| Cluster 11 | 2854 | RiPP -like | unknown |  |  |
| **RHF15** |  |  |  |  |  |
| Cluster 1 | 40888 | T3PKS | unknown |  |  |
| Cluster 2 | 20849 | Terpene | unknown |  |  |
| Cluster 3 | 82212 | NRPS | fengycin | 100 | *B. velezensis FZB42* |
| Cluster 4 | 114771 | Hybrid PKS/NRPS | bacillaene | 100 | *B. velezensis FZB42* |
| Cluster 5 | 20803 | Terpene | unknown |  |  |
| Cluster 6 | 41418 | Other | bacilysin | 100 | *B. velezensis FZB42* |
| Cluster 7 | 21611 | RiPP (Thiopeptide) | subtilosin A | 100 | *B. subtilis subsp. spizizenii ATCC 6633* |
| Cluster 8 | 20746 | CDPS | unknown |  |  |
| Cluster 9 | 49741 | NRPS | bacillibactin | 100 | *B. subtilis subsp. subtilis str. 168* |
| Cluster 10 | 65391 | NRPS (Lipopeptide) | surfactin | 82 | *B. velezensis FZB42* |
| Cluster 11 | 24457 | RiPP (Lanthipeptide) | subtilomycin | 100 | *B. subtilis* |
| **RHFS10** |  |  |  |  |  |
| Cluster 1 | 41097 | T3PKS | unknown |  |  |
| Cluster 2 | 21898 | Terpene | unknown |  |  |
| Cluster 3 | 128639 | NRPS | fengycin | 100 | *B. velezensis FZB42* |
| Cluster 4 | 114812 | Hybrid PKS/NRPS | bacillaene | 100 | *B. velezensis FZB42* |
| Cluster 5 | 41418 | Other | bacilysin | 100 | *B. velezensis FZB42* |
| Cluster 6 | 21613 | RiPP (Thiopeptide) | subtilosin A | 100 | *B. subtilis subsp. spizizenii ATCC 6633* |
| Cluster 7 | 20746 | CDPS | unknown |  |  |
| Cluster 8 | 49742 | NRPS | bacillibactin | 100 | *B. subtilis subsp. subtilis str. 168* |
| Cluster 9 | 81352 | Hybrid PKS/NRPS | zwittermicin A | 18 | *B. cereus* |
| Cluster 10 | 20788 | Terpene | unknown |  |  |
| Cluster 11 | 65394 | PKS | macrolactin H | 90 | *B. velezensis FZB42* |
| Cluster 12 | 22952 | RiPP (Head-to-tailcyclized peptide) | sporulation killing factor | 100 | *B. subtilis subsp. subtilis str. 168* |
| **RHFS18** |  |  |  |  |  |
| Cluster 1 | 105749 | Hybrid PKS/NRPS | Difficidin | 100 | *B. velezensis FZB42* |
| Cluster 2 | 40665 | T3PKS | unknown |  |  |
| Cluster 3 | 20138 | Terpene | unknown |  |  |
| Cluster 4 | 120565 | NRPS | fengycin | 93 | *B. velezensis FZB42* |
| Cluster 5 | 109609 | Hybrid PKS/NRPS | bacillaene | 100 | *B. velezensis FZB42* |
| Cluster 6 | 88235 | PKS | macrolactin H | 100 | *B. velezensis FZB42* |
| Cluster 7 | 20740 | Terpene | unknown |  |  |
| Cluster 8 | 41244 | Saccharide | butirosin A/ butirosin B | 7 | *B. circulans* |
| Cluster 9 | 65410 | NRPS (Lipopeptide) | surfactin | 82 | *B. velezensis FZB42* |
| Cluster 10 | 29736 | LAP, thiopeptide | unknown |  |  |
| Cluster 11 | 8 | Other | bacilysin | 100 | *B. velezensis FZB42* |
| Cluster 12 | 51794 | NRPS | bacillibactin | 100 | *B. subtilis subsp. subtilis str. 168* |
